# Cone-beam CT delta-radiomics to predict genitourinary toxicities and international prostate symptom of prostate cancer patients: a pilot study

**DOI:** 10.1038/s41598-022-24435-8

**Published:** 2022-11-22

**Authors:** Rodrigo Delgadillo, Benjamin O. Spieler, Anthony M. Deana, John C. Ford, Deukwoo Kwon, Fei Yang, Matthew T. Studenski, Kyle R. Padgett, Matthew C. Abramowitz, Alan Dal Pra, Radka Stoyanova, Nesrin Dogan

**Affiliations:** 1 Department of Radiation Oncology, University of Miami Miller School of Medicine, 1475 NW 12th Ave, Miami, FL 33136 USA; 2 Department of Biomedical Engineering, University of Miami, Miami, FL USA; 3 Center for Clinical and Translational Sciences, The University of Texas Health Science Center at Houston, Houston, TX USA

**Keywords:** Cancer imaging, Tumour biomarkers

## Abstract

For prostate cancer (PCa) patients treated with definitive radiotherapy (RT), acute and late RT-related genitourinary (GU) toxicities adversely impact disease-specific quality of life. Early warning of potential RT toxicities can prompt interventions that may prevent or mitigate future adverse events. During intensity modulated RT (IMRT) of PCa, daily cone-beam computed tomography (CBCT) images are used to improve treatment accuracy through image guidance. This work investigated the performance of CBCT-based delta-radiomic features (DRF) models to predict acute and sub-acute International Prostate Symptom Scores (IPSS) and Common Terminology Criteria for Adverse Events (CTCAE) version 5 GU toxicity grades for 50 PCa patients treated with definitive RT. Delta-radiomics models were built using logistic regression, random forest for feature selection, and a 1000 iteration bootstrapping leave one analysis for cross validation. To our knowledge, no prior studies of PCa have used DRF models based on daily CBCT images. AUC of 0.83 for IPSS and greater than 0.7 for CTCAE grades were achieved as early as week 1 of treatment. DRF extracted from CBCT images showed promise for the development of models predictive of RT outcomes. Future studies will include using artificial intelligence and machine learning to expand CBCT sample sizes available for radiomics analysis.

## Introduction

Prostate cancer (PCa) is the most common malignancy among men and the second-leading cause of cancer-related mortality in the United States (US)^1^. With 3.1 million PCa survivors in the US, acute and late side effects of PCa treatment impact a significant proportion of US men^2^. Definitive radiotherapy (RT), a primary intervention for intermediate and high-risk PCa, aims to limit treatment-related side effects and preserve patient quality of life (QOL) while delivering curative dose to the prostate^3–6^. Efforts to minimize toxicity to nearby organs at risk (OAR), mainly gastrointestinal and genitourinary (GU) tissues, have established intensity-modulated radiotherapy (IMRT) as the standard external beam technique for RT of PCa^7–10^. Modern image-guided RT (IGRT) with linac-mounted cone-beam CT (CBCT) improves the therapeutic index through enhanced visualization of the prostate prior to daily treatment, allowing for reduction of planning target volume (PTV) margins and sparing of adjacent OAR^6,7,11,12^. Despite innovations in RT techniques, modalities and fractionation, overall patient QOL suffers compared to men without PCa in part due to RT-related side effects^13^. As a result, prevention and alleviation of symptoms that impact QOL during and after RT is a priority for PCa survivorship research and clinical practice^14,15^. Published data support an association between acute and late post-RT toxicity among PCa patients, with early QOL decline linked to long-term dissatisfaction^16,17^. Studies suggest that acute toxicity and early decline in QOL scores can be used to identify patients who may benefit from personalized supportive care intended to mitigate high risk of late toxicity^17,18^. Accordingly, early prediction of acute and subacute side effects, as well as compromised QOL, can prompt interventions and preventative strategies that benefit PCa survivorship^18,19^.

Radiomics, an analytic technique that extracts quantitative characteristics from medical imaging, has been incorporated into predictive models in diverse cancers including PCa^20–27^. Delta-radiomics is a version of radiomics applied to medical imaging at multiple time points to identify changes in tissue biology typically in response to a therapeutic intervention. Increasing evidence suggests that radiomic features extracted from imaging platforms such as computed tomography (CT), ultrasound, and magnetic resonance imaging (MRI) may be useful as non-invasive biomarkers to predict cancer treatment response and prognosis. Recent publications analyzing diagnostic imaging before and after PCa RT have identified radiomic features that may be predictive of patient outcomes, but do not describe tissue response during RT itself^20–27^. Few previous radiomics studies investigated the use of daily CBCT setup images for predicting patient response^28,29^. To our knowledge, no prior radiomics studies of PCa have analyzed daily CBCT images. In this study, we hypothesized that delta-radiomics of daily patient CBCT set-up images during definitive IMRT of PCa can predict acute and subacute RT-related toxicities, providing clinicians with the opportunity to implement preventative strategies and early interventions that benefit PCa survivorship. This is a pilot study to test the feasibility of predicting acute GU toxicity, sub-acute GU toxicity, and change in International Prostate Symptom Scores (IPSS) using daily CBCT-based delta-radiomics acquired during definitive RT of PCa.

## Materials and methods

### Patient population

Fifty patients enrolled in institutional review board (IRB)-approved protocols for the treatment of PCa were selected for this study. The ethical approval for this study was obtained from the University of Miami Institutional Review Board (IRB). Written informed consent was obtained from all patients in this study. The data was retrospectively collected and analyzed. All methods undertaken in this work were carried out in accordance with the relevant guidelines and regulations. Characteristics of the patient cohort are summarized in Supplementary Table S1. To maintain consistency in imaging quality and reduce variability between different kV imaging devices available in the facility several constraints were placed on the patients selected for this study. Only PCa patients who received definitive volumetric modulated radiation therapy (VMAT) on a TrueBeam linear accelerator (Varian Medical Systems, Palo Alto, CA) with daily CBCT were included in the study. Raw projection data was exported from the treatment machines and later reconstructed using image reconstruction software to maintain consistent reconstruction parameters for all the images. More details on image reconstruction are described in the following section. Patients with body mass index (BMI) > 40 or intrapelvic metal prostheses were excluded due to X-ray hyper attenuation and metal streaking artifacts, respectively. CBCT images were analyzed from the daily scans throughout the entire course of treatment.

### Imaging characteristics and reconstruction

All clinical CBCT images used in this study had the same image size (512 × 512 pixels), pixel size (0.9 mm), slice thickness (2 mm), and FOV (465 mm). The tube voltage (125 kVp) and tube current–time product (1073–1074 mAs) were consistent for all CBCT scans.

sCBCT refers to CBCT images reconstructed using standard filtered back-projection reconstruction. iCBCT refers to CBCT images reconstructed using an iterative reconstruction algorithm that employs a scatter correction method that estimates scatter in X-ray projection images by solving the linear Boltzmann transport equation and statistical iterative reconstruction for final-pass image reconstruction^30^. For all patients, raw projections of both sCBCT and iCBCT images were collected to reconstruct CBCT images using the reconstruction method of choice (sCBCT or iCBCT) post-treatment. A research image reconstruction software (iTools, Varian Medical Systems, Palo Alto, CA, USA) was used to generate either sCBCT or iCBCT image sets for each patient. Daily CBCT raw projections were reconstructed utilizing eight combinations of various reconstruction algorithms, convolution filters, and noise suppression filters (Table 1). One aim of the iterative reconstruction algorithm is to minimize the variation in voxel intensity in the final reconstruction. The degree to which this variation is minimized is referred to as the noise suppression. While noise suppression is only available in iCBCT, different convolution filters can be applied to both iCBCT and sCBCT. Convolution filters can affect image spatial frequency characteristics by convolving the image with a kernel. Though the aim of convolution filters and noise suppression is to improve image quality in one way or another, there are tradeoffs. Smooth convolution filters can reduce noise at a loss of resolution. Very high noise suppression will reduce noise at a loss of contrast. Delgadillo et al. analyzed the repeatability and reproducibility of CBCT-based radiomic features for PCa patients receiving RT^31^ and found that reconstruction and preprocessing parameters that improve feature repeatability often decrease reproducibility^31^. Reconstruction and preprocessing paraments that strike a balance between repeatability and reproducibility are recommended. Considering tradeoffs between image filters and image quality and the findings from Delgadillo et al.^31^, CBCT images in this study were reconstructed using *Very Low* noise suppression, when applicable, and *Sharp* convolution filter. Since CT has higher image quality and has been shown to be useful for modeling and predicting outcome^25^, it would be useful if some predictive feature were reproducible to daily CBCT to exploit changes during treatment that are predictive to patient outcome. However, the reconstruction parameters used in the clinic are typically *Medium* noise suppression, when applicable, and standard convolution filter. The default clinical image reconstruction settings were also included to compare the delta-radiomics feature model performance for CBCT with reconstruction parameters more typically used in other clinics. An example of a reconstructed prostate CBCT image using the different reconstruction parameters considered in this work is shown in Supplementary Fig. 1.

**Table 1:** Reconstruction and pre-processing parameters considered in this work. ‘1’ means Collewet Normalization was applied and ‘0’ means Collewet Normalization was not applied.

Abbreviation	Reconstruction algorithm	Convolution filter	Noise suppression	Quantization algorithm	Collewet normalization
iCBCT-Sharp-VeryLow-Llo-1	iCBCT	Sharp	Very low	Lloyd-Max	1
iCBCT-Sharp-VeryLow-Uni-0	iCBCT	Sharp	Very low	Uniform	0
iCBCT-Std-Medium-Llo-1	iCBCT	Standard	Medium	Lloyd-Max	1
iCBCT-Std- Medium-Uni-0	iCBCT	Standard	Medium	Uniform	0
sCBCT-Sharp-Llo-1	sCBCT	Sharp		Lloyd-Max	1
sCBCT-Sharp-Uni-0	sCBCT	Sharp		Uniform	0
sCBCT-Std-Llo-1	sCBCT	Standard		Lloyd-Max	1
sCBCT-Std-Uni-0	sCBCT	Standard		Uniform	0

Image processing, as defined by IBSI, includes procedures such as interpolation, range-re-segmentation, discretization (quantization), and image filtering^32^. The quantization algorithm refers to how the image intensities are quantized into discrete bins. Collewet normalization is a normalization where the gray levels of the ROI are normalized from the range of $[{\mu }_{R}-3{\sigma }_{R},{\mu }_{R}+3{\sigma }_{R}]$ where ${\mu }_{R}$ was the mean and ${\sigma }_{R}$ was the standard deviation of the ROI gray levels^33^. The IBSI notation refers to this type of Collewet normalization as a re-segmentation method RS:3σ 7ACA^32^. The work by Delgadillo et al. suggested that the Lloyd-Max quantization algorithm and Collewet normalization provided the best balance between repeatability and reproducibility of CBCT-based radiomic features^31^. The Lloyd-Max quantization algorithm is an algorithm where bin levels are assigned in a way that minimizes quantization error^34,35^. For this reason, the Lloyd-Max quantization algorithm with Collewet normalization (Llo-1) was applied to the reconstructed daily CBCT images. The most basic quantization algorithm is the Uniform quantization algorithm that defines bins by evenly distributing them from lowest to highest grey level. In the IBSI notation the uniform quantization is known as an intensity discretization with fixed bin number^32^. The Uniform quantization algorithm without Collewet normalization (Uni-0) was also applied to the reconstructed daily CBCT images to generate a parallel data set to the reconstructions with Llo-1. The reason for also considering Uni-0 was to analyze the capability of producing models using the most basic form of image quantization. Previous work on the CBCT-based radiomics of prostate showed that the number of quantization bins did not have a large effect on the repeatable and reproducibility of radiomic features, but the most repeatable and reproducible radiomic features typically did have 256 quantization bins^31^. Thus, the number of quantization bins was set to 256, or 8 bits to be consistent.

### Prostate contours

The prostate served as the region of interest (ROI) for the radiomic feature extraction. Published studies have found that patients with large prostates (> 50 cc) develop significantly more acute and late urinary toxicity than patients with small prostates after EBRT, most likely due to an inverse correlation between the volume of inflamed prostatic tissue and urethral patency^36,37^. Importantly, this increased GU toxicity in patients with large prostate volumes is considered a direct result of prostate RT, and not a reflection of differences in underlying urinary function or bladder dosimetry^36^. The urethra is a distensible luminal organ acutely sensitive to extramural inflammation, and tissue volume changes arising anywhere within the prostate can impact lower urinary tract outflow. Based on these considerations, in our study plan the entire prostate including the urethra was segmented for radiomic analysis and modeled for prediction of intra- and post-RT GU adverse events. A team of radiation oncologists with expertise in prostate cancer RT delineated the prostate on the planning CT (pCT) with the aid of MRI. First, prostate contours were propagated from the pCT to the daily CBCTs (both sCBCT and iCBCT) by applying the shifts from rigid registration used for daily patient setup. The prostate may change in volume or shape between the planning CT and daily CBCT, and other inconsistencies due to registration errors in the prostate contour may occur. In order to manage these discrepancies, a radiation oncologist reviewed the daily CBCT prostate contours and when needed, corrected the contours directly on the CBCTs utilizing imaging software (MIM, ver. 6.8.1, MIM Software Inc., Cleveland, OH). Gold fiducial artifacts were removed from prostate contours on CBCT images prior to RF extraction using the algorithm described in Delgadillo et al.^31^. The fiducial artifact removal algorithm sets an artifact threshold defined with range $[{\mu }_{AL}-3{\sigma }_{AL},{\mu }_{AL}+3{\sigma }_{AL}]$ where ${\mu }_{AL}$ is the mean and ${\sigma }_{AL}$ is the standard deviation of the voxel intensity levels on layers not containing fiducial artifacts. On layers containing fiducial artifacts, a mask was generated by defining a circle of 5 mm centered on the fiducial marker, including pixels that exceeded the artifacts threshold. Typically, metal streaking artifacts in prostate radiate outwards from the center of the fiducial. To capture this aspect, masks were generated to overlayed pixel mask lines between the distal artifact pixels to the center of the fiducial. An example of the fiducial artifact removal is shown in Supplementary Fig. 2. Fiducial artifact removal resulted in a mean percent decrease of 18% in the prostate volume. Though information is lost, removal of fiducial artifacts allows for the use of 3D radiomics. A limitation of other radiomics studies in the presence of fiducial artifacts is that they only consider 2D radiomics on one slice that was chosen because it contained no fiducial artifacts^38^.

### Description of delta-radiomic features

Forty-two RFs were extracted from prostate contours on daily CBCT images. Forty-two RFs were considered because they represent RFs from the most commonly used classes. Moreover, many more radiomic features are possible though research has demonstrated that many radiomic features are intercorrelated^39,40^. RFs were calculated using the MATLAB (MATLAB, ver. 2020b, Math-Works Inc., Natick, MA) “Radiomics” package developed by Vallieres, et al. in combination with in-house code to extract 3D bitmaps of the ROI using the DICOM structure files from the CT DICOM files^41^. Textural features were calculated from five RF classes including gray-level co-occurrence matrices (GLCM), Neighborhood Gray-Tone Difference Matrix (NGTDM), gray-level run length matrices (GLRLM), and gray-level size zone matrices (GLSZM), and first order statistical features. These features are described in detail in Delgadillo et al*.*, including the Image Biomarker Standardization Initiative (IBSI) code equivalent^31,42^. The full list of RFs along with their IBSI code equivalent are shown in Supplementary Table S3. While forty RFs were IBSI compliant, NGTDM Coarseness and Strength were not IBSI-compliant. Those definitions can be found in Amadasun and King^43^. In addition to these forty-two features, volume-normalized versions NGTDM Strength, NGTDM Busyness, NGTDM Coarseness, GLSZM GLN, GLRLM GLN, and GLRLM RLN were also considered.

The prostate volume can impact radiomic feature analysis because it is known to correlate with GU toxicities^36,37^. Other researchers have also noted that a region of interest’s volume can have a strong correlation with radiomics features and can thus have a confounding effect on radiomic analysis^44–46^. Three approaches were used in this study to account for the effects of prostate volume on radiomic analysis. First, the extracted images were isotopically resampled to a 1 mm voxel size in order to reduce voxel size dependence of radiomic features^45,46^. Second, volume normalizations (VN) from Shafiq-ul-Hassan et al*.*^45^ was used for NGTDM Strength, GLSZM GLN, GLRLM GLN, and GLRLM RLN volume normalizations (VNF) from Fave et al.^44^ were used for NGTDM Busyness and NGTDM Coarseness. Third, prostate volume was included the RF space along with the other previously mentioned RFs.

To make the changes in RFs between patients more comparable, RFs were normalized to the first fraction. The delta-radiomics feature of a given RF (DRF) was defined as:

$$DRF=\frac{R{F}_{N}-R{F}_{1}}{|R{F}_{1}|},$$

where $R{F}_{N}$ is the RF value of the Nth fraction and $R{F}_{1}$ is the RF value of the 1st fraction.

### Using biologically effective dose (BED) to bin radiomic features from different fractionation schedules

**Figure 1:**
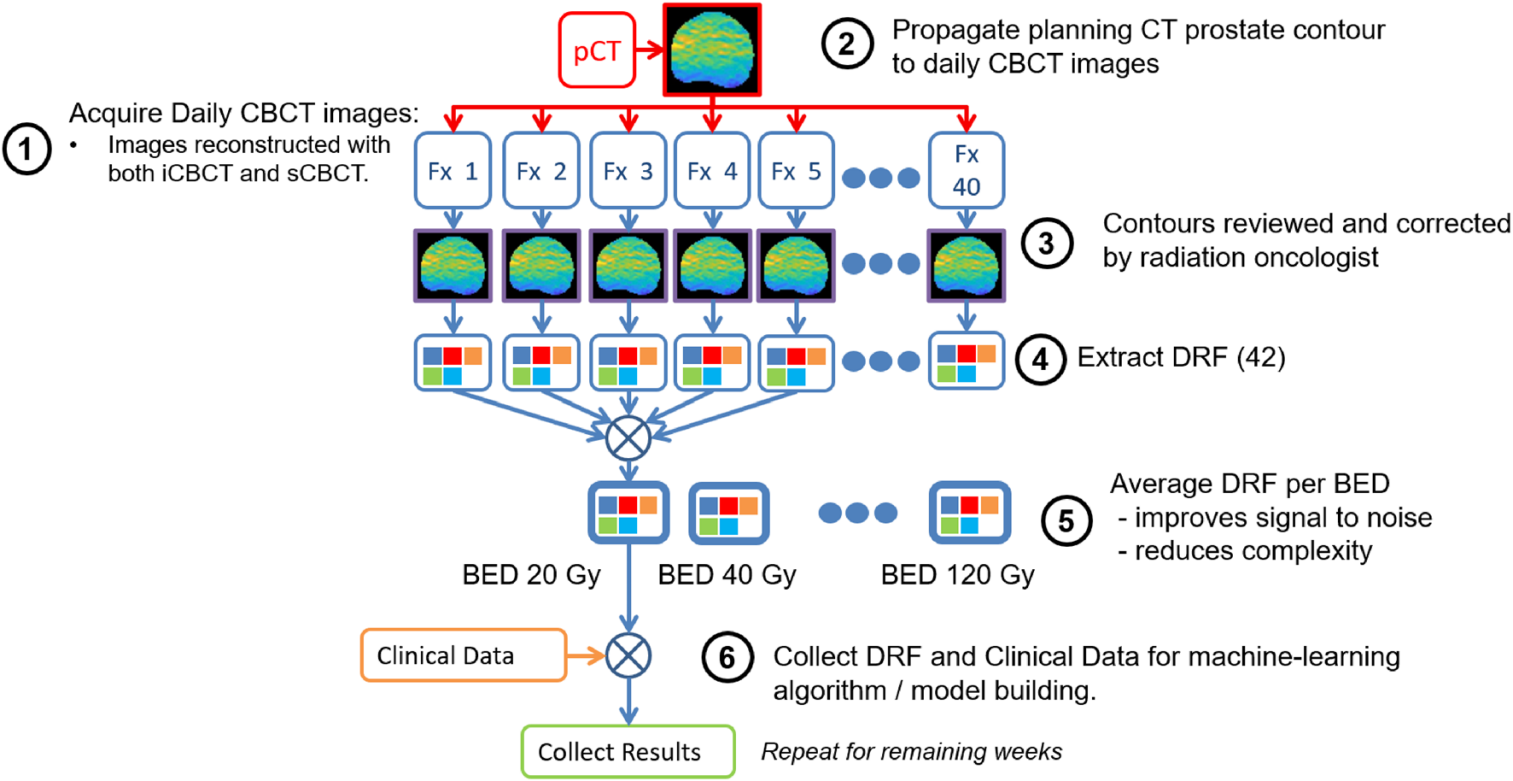
Extraction of delta-radiomic features **(**DRF) from prostate daily CBCT images for analysis and model building.

The patients considered in this study were treated with a variety of dose fractionation schedules as summarized in Supplementary Table S1. Time points were considered on a BED binned basis to account for the different schedules. BED was calculated using the formula:

$$BED=nd\left(1+\frac{d}{\alpha /\beta }\right),$$

where $n$ is the number of fractions, $d$ the dose per fraction, and $\alpha /\beta$ is the linear-quadratic ratio for the specified tissue. An $\alpha /\beta$ = 3 was used to account for dose delivered to the prostate for the BED calculation^47^. DRFs were averaged per accumulated BED bins between 20 to 120 Gy BED bins with a bin width of 20 Gy. A 20 Gy BED bin width was chosen as a compromise between the standard 80 Gy in 40 fraction treatment, which includes 6 fractions per BED bin, and the moderately hypofractionated 70.2 Gy in 26 fraction treatment, which includes 4 fractions per BED bin. Thus, a 20 Gy BED bin roughly represents one week of treatment. The workflow for the extraction of delta-radiomics features along with BED bin averaging is shown in Fig. 1.

### Clinical endpoints

**Figure 2:**
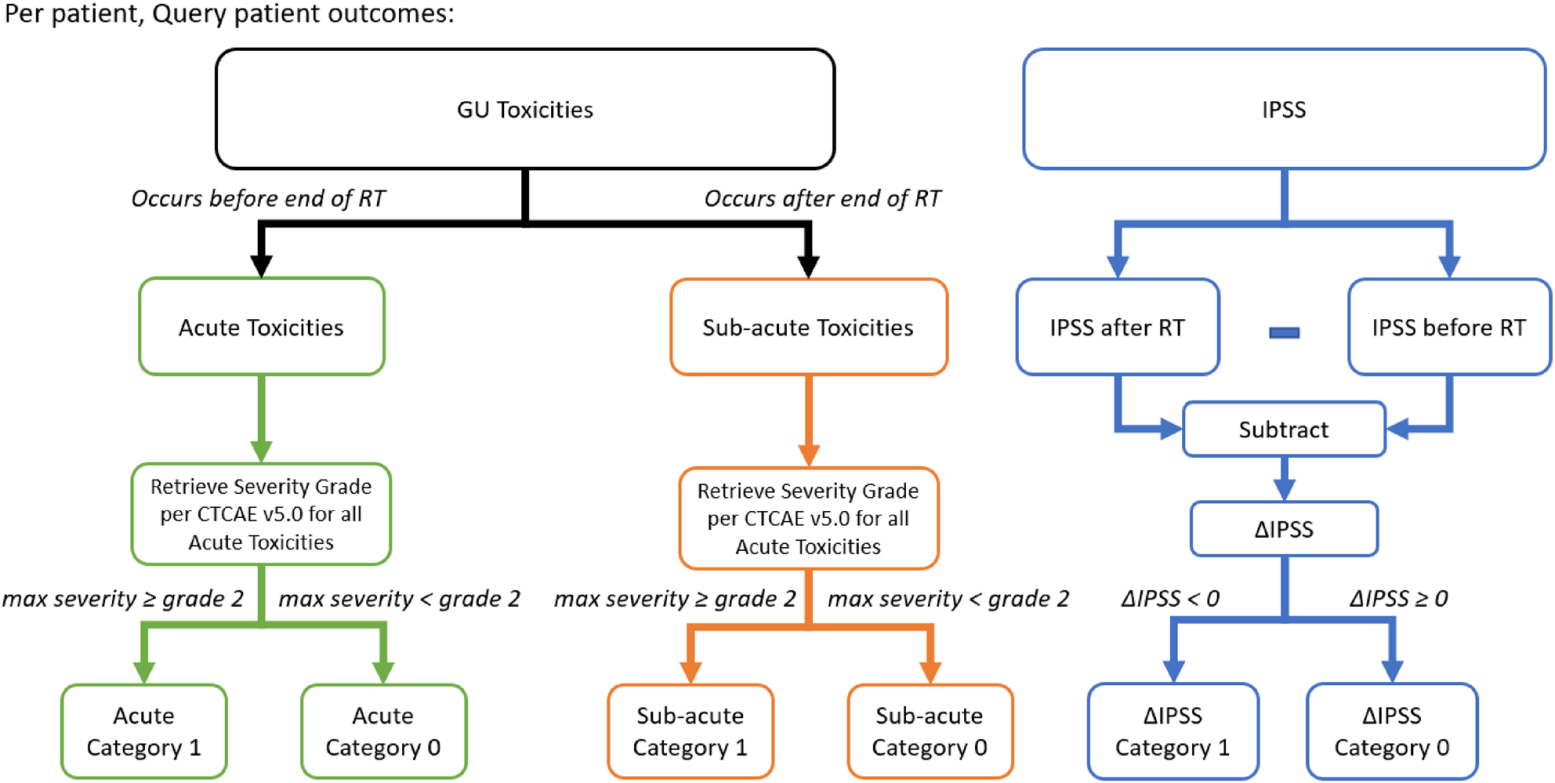
Visual representation of categorization of clinical endpoints for this study.

Clinical endpoints considered were acute and sub-acute GU adverse events graded per CTCAE v5.0 and total International Prostate Symptom Score^48,49^ (IPSS). Acute GU toxicities were defined as toxicities that occurred before the end of RT and sub-acute GU toxicities were toxicities that occurred after RT. GU toxicities considered were frequency, nocturia, dysuria, urgency, urinary obstructive symptoms, and incontinence. A summary of the percentages of events for the outcomes and BED levels is shown in Supplementary Table S2. A visual representation of the categorization of the clinical endpoints is shown in Fig. 2.

#### GU toxicities

Logistic regression models were built by categorizing GU toxicities based on the max severity after their respective time point. For example, only toxicities that occurred after the patient accumulated 20 Gy was considered for the 20 Gy BED bin. For sub-acute GU toxicities, only toxicities occurring after the end of treatment were considered. Thus, the sub-acute GU toxicities endpoint did not change with the BED bin. For the logistic regression, GU toxicities were further categorized by their CTCAE v5.0 grade:

- Category 1: Maximum severity ≥ grade 2
- Category 0: Maximum severity < grade 2

#### IPSS

From a clinical perspective, the change in IPSS due to treatment was the relevant metric to describe how a patients urinary related quality of life changed due to the radiation therapy. Consequently, the ∆IPSS is defined:

$$\Delta IPSS=IPS{S}_{f}-IPS{S}_{i},$$

where $IPS{S}_{f}$ is the patient’s final score after treatment and $IPS{S}_{i}$ is the patient’s initial score before treatment. For the logistic regression, ∆IPSS were categorized as:

- Category 1: ∆IPSS < grade 0
- Category 0: ∆IPSS ≥ grade 0

A low IPSS score indicates that the patient has few to no occurrences of urinary-related symptoms on the IPSS questionnaire and a high IPSS score indicates more occurrences of lower urinary tract symptoms (LUTS). Consequently, a positive ∆IPSS indicates a detriment to the patient and a negative ∆IPSS indicates an improvement to the patient.

### Model building and data analysis

**Figure 3:**
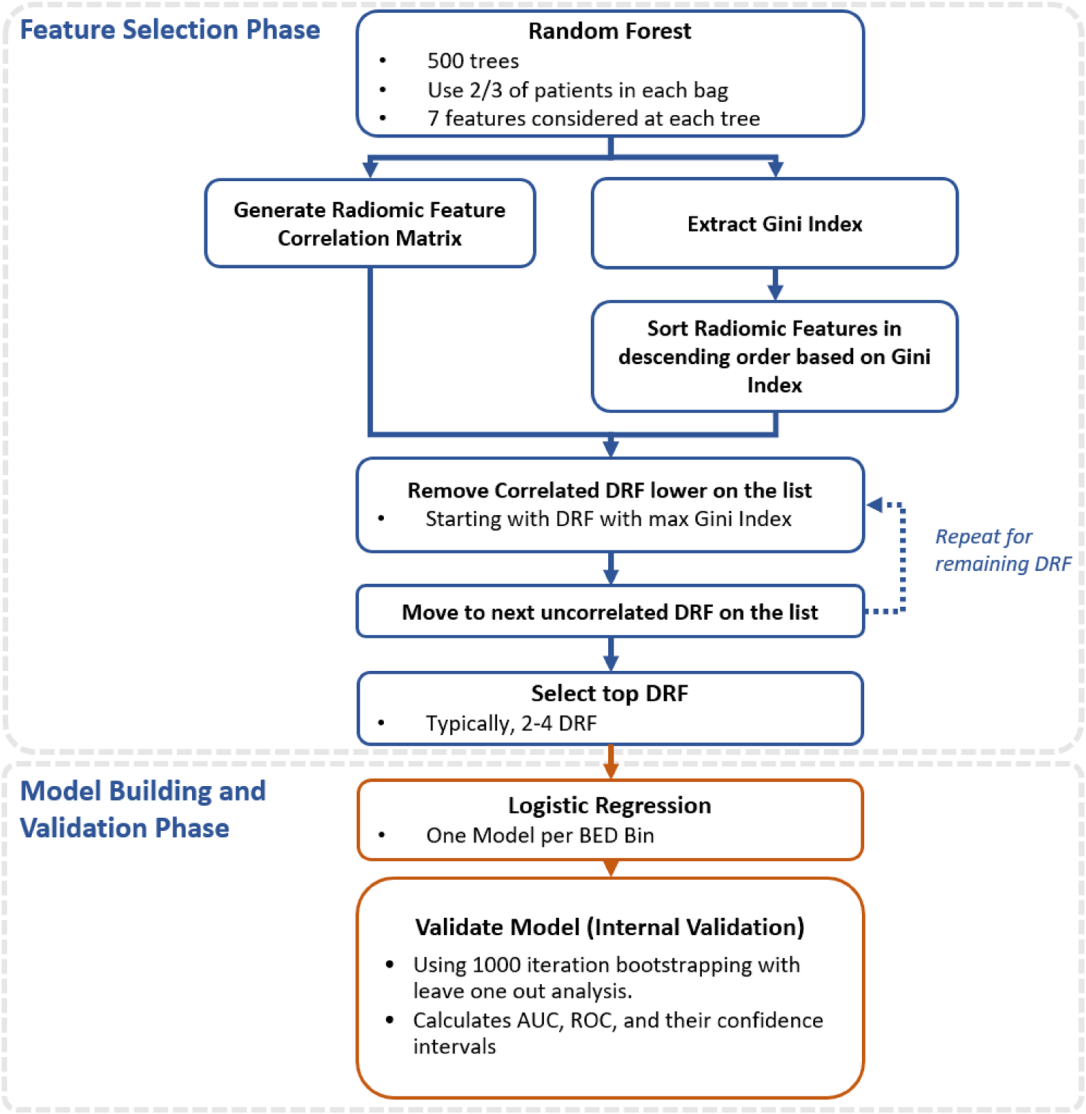
DRF model and analysis workflow.

Delta-radiomics models of acute GU toxicities, sub-acute GU toxicities, and ∆IPSS were generated using logistic regression. Model building and data analysis were performed using a statistics and machine learning toolbox from scientific computation software (MATLAB, ver. 2020b, Math-Works Inc., Natick, MA). Models were built for every BED bin to analyze model predictive properties as a function of time. The model building and data analysis workflow are shown in Fig. 3.

The feature selection phase is the first phase of the model building and data analysis workflow. The concept is to ultimately choose the DRF that will go into the final models. All BED bins were considered together as one cohort in the feature selection phase to keep the DRF allowed in the model to be consistent across all BED bins. One could consider a scenario where feature selection is performed independently for each BED bin, but it was decided that this would add more uncertainty and make it more difficult to compare model performance as function of BED.

**Figure 4:**
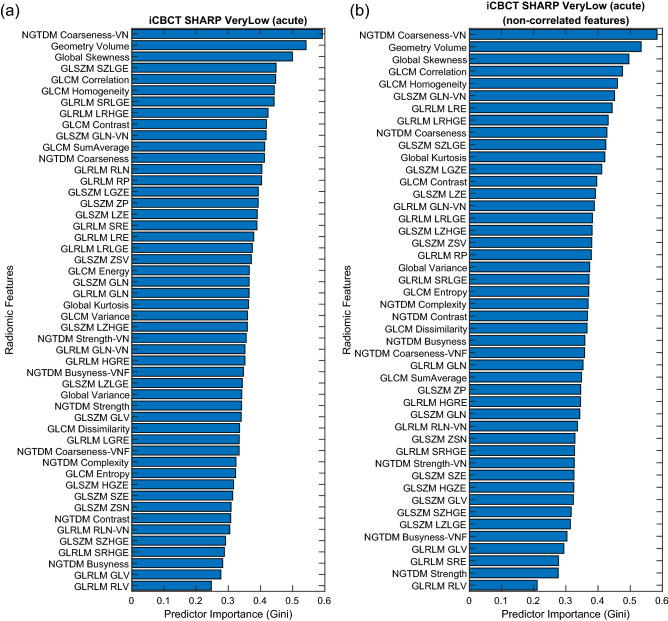

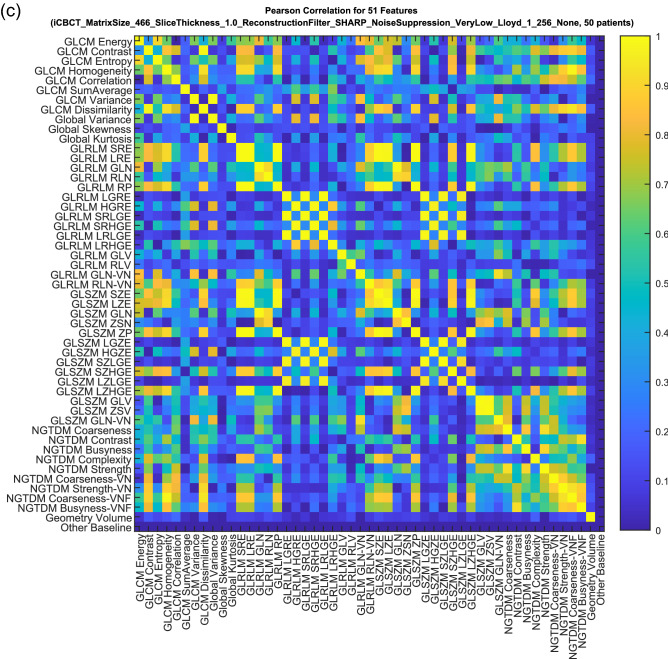
During the feature selection phase, correlated DRF were filtered out from (**a**) the initial predictor importance ranking to produce (**b**) the final predictor importance ranking after removing the lower ranked correlated features using (**b**) the Correlation Matrix between the DRF. This example corresponds to iCBCT-SHARP-VeryLow-Llo-1 for Sub-Acute GU toxicity.

A Random Forest approach was used for feature selection^50,51^. Feature selection was considered independently for acute toxicities, sub-acute toxicities, and IPSS. The random forest contained 500 trees and two-thirds of the patients in each bag. At each tree, 7 radiomic features were used to prevent overfitting using the convention that the number of radiomic features at each tree should be limited to the square root of the total number of features in the feature space^52^. Often for modelling with multiple variables, some input predictor variables will turn out to be more relevant than other predictor variables. One way to measure predictor performance is the Gini index. The Gini index is estimated by first accumulating the changes in the risk due to splits on every predictor and by then dividing the sum of the number of branch nodes^52^. Consequently, the radiomic features were ranked using the Gini index to select the most important delta-radiomics features. An example of the Gini index is shown in Fig. 4.

In previous work by Traverso et al., 80% of 843 radiomics features they considered were inter-correlated and 30% of the radiomic features correlated with volume^39^. This could lead to radiomic features that do not contribute very much new, if any, information in terms of building the model. Consequently, cross-radiomic feature correlation matrices were calculated the Pearson correlation between radiomic features for all patients and combination of radiomic features, including volume. Correlation matrices were built for every reconstruction and preprocessing method considered in this work. An example of the radiomic feature correlation matrix is shown in Fig. 4. A cross-radiomic feature correlation threshold of greater than 0.8 was used to determine if a given radiomic feature was strongly correlated with another^53^. The list of radiomic features in the Gini ranking was filtered by only keeping the radiomic feature with the highest Gini index in cases where a set of radiomic features correlated strongly with each other. Thus, correlated radiomic features were removed from consideration for feature selection. Then, the most important radiomic features were selected by analyzing the Gini index of non-correlated radiomic feature, as shown in the example in Fig. 4. Starting from the highest-ranked radiomic feature, a search was performed to find radiomic features with high importance. A maximum of seven DRF were allowed for feature selection to prevent over fitting.

The DRF that passed the feature selection phase were then used for model building. Each BED bin was modeled independently with the constraint that they used the same selected DRF. However, the different clinical endpoints and reconstruction and preprocessing parameters were considered in parallel workflows independently. The clinical endpoints were modeled using logistic regression since the clinical endpoints considered in this generally could be divided into two categories.

A leave one out cross validation with 1000 bootstrapping iterations was used. The benefit of the leave one out cross validation approach is that all the data could go into the training of model, but the downside of this approach is that there is no external validation^54^. However, given the limitations of available patient sample size, due to patient selection requirements described earlier, it was best approach to use only internal validation for this pilot study. A future work should allow for more patients and to utilize external validation set. The benefits of using a leave one analysis over other cross validation methods that assign a greater percentage of data are that more data is included in the training set. Finally, the area under the curve (AUC) and their confidence intervals and receiving operating characteristic curves (ROC) were calculated. In this work, we considered Delta-radiomics models with AUC of 0.7–0.8 as moderate performing, and AUC greater than 0.8 as strong performing. For IPSS models only, the baseline IPSS, which was acquired prior to radiotherapy, was included in the feature space as well.

## Results

**Figure 5:**
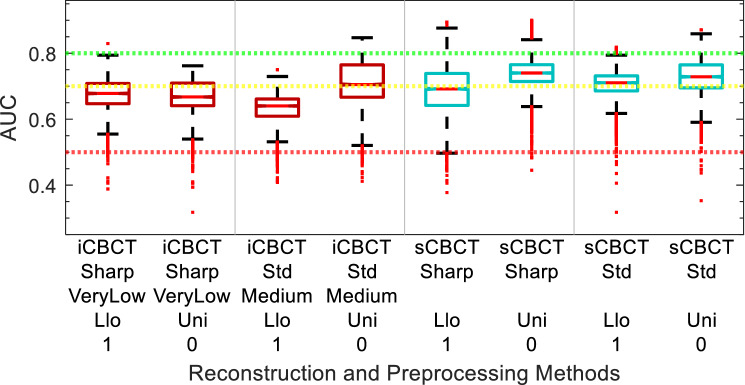
Acute GU Toxicity AUC of delta-radiomic models vs Reconstruction and Preprocessing methods. The AUC collected over 1000 iterations of the leave-one analysis and over all BED bins.

**Figure 6:**
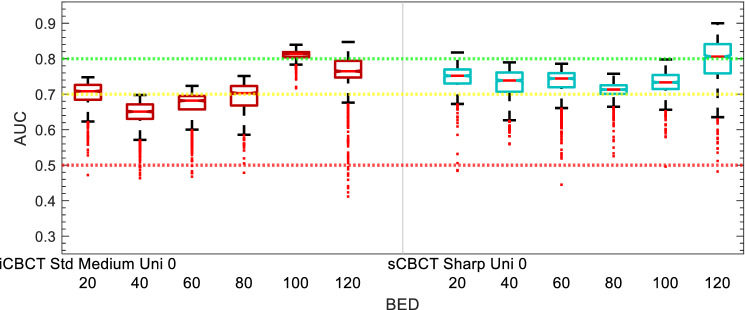
Acute GU Toxicity AUC of delta-radiomic models vs BED bins. The AUC collected over 1000 iterations of the leave-one analysis.

**Figure 7:**
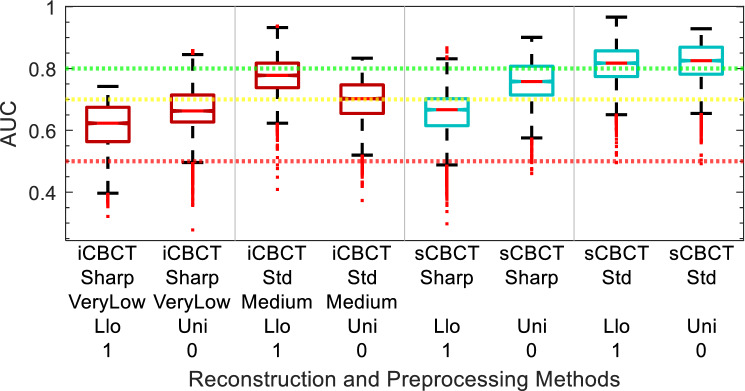
Sub-Acute GU Toxicity AUC of delta-radiomic models vs Reconstruction and Preprocessing methods. The AUC collected over 1000 iterations of the leave-one analysis and over all BED bins.

**Figure 8:**
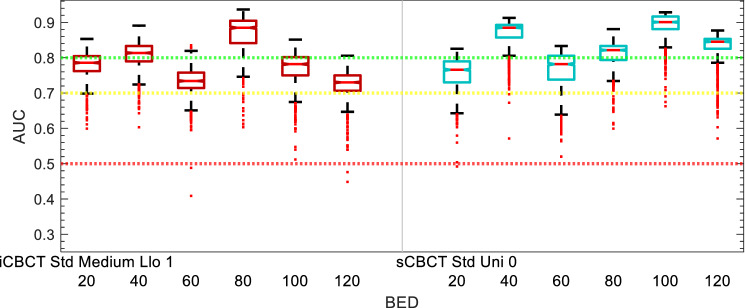
Sub-Acute GU Toxicity AUC of delta-radiomic models vs BED bins. The AUC collected over 1000 iterations of the leave-one analysis.

**Figure 9:**
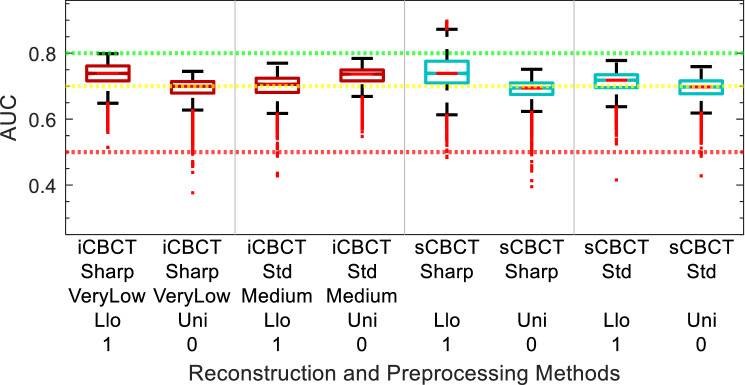
∆IPSS AUC of delta-radiomic models vs Reconstruction and Preprocessing methods. The AUC collected over 1000 iterations of the leave-one analysis and over all BED bins.

**Figure 10:**
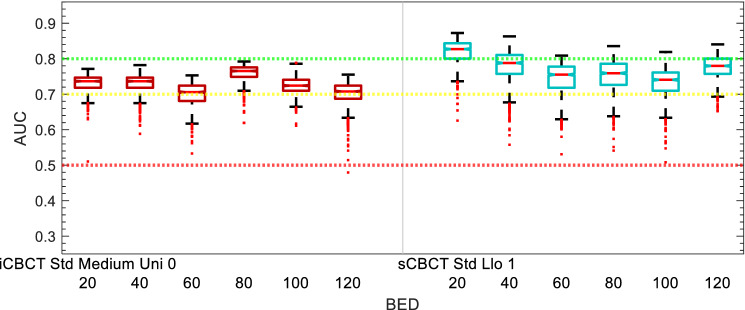
∆IPSS AUC of delta-radiomic models vs BED bins. The AUC collected over 1000 bootstrapping iterations using the leave-one analysis.

AUC performance of the models are shown in Figs. 5, 6, 7, 8, 9 and 10. The performance of the models varied due to several factors including the different clinical endpoints, the reconstruction algorithm, the feature pre-processing method, and the accumulated BED. The results of the radiomic feature selection are shown in Table 2. Both the number of selected features and the specific features selected varied depending on the clinical endpoint and reconstruction and pre-processing method.

**Table 2:** Summary of the top DRF found after the feature selection phase organized by clinical endpoint and reconstruction and pre-processing method. Asterisks were placed on the iCBCT and sCBCT reconstruction and pre-processing methods with the best performing AUC in their respective categories.

Clinical endpoint	Reconstruction and pre-processing method	N	Selected features
Acute	iCBCT SHARP VeryLow Llo 1	6	GLCM Correlation, GLCM Homogeneity, GLSZM GLN-VN, Global Skewness, NGTDM Coarseness-VN, Prostate Volume
Acute	iCBCT SHARP VeryLow Uni 0	3	GLCM Correlation, NGTDM Coarseness-VN, Prostate Volume
Acute	iCBCT STD Med Llo 1	4	GLCM Contrast, GLCM Dissimilarity, GLSZM LZHGE, Prostate Volume
Acute	iCBCT STD Med Uni 0*	4	GLSZM LZHGE, NGTDM Coarseness-VN, NGTDM Strength, Prostate Volume
Acute	sCBCT SHARP Llo 1	6	GLCM Energy, GLRLM LRHGE, GLSZM GLN-VN, Global Kurtosis, Global Skewness, NGTDM Busyness
Acute	sCBCT SHARP Uni 0	5	GLRLM GLN-VN, GLSZM GLN, GLSZM GLN-VN, NGTDM Coarseness, Prostate Volume
Acute	sCBCT STD Llo 1	4	GLSZM GLN, GLSZM GLN-VN, Global Kurtosis, Prostate Volume
Acute	sCBCT STD Uni 0*	6	GLRLM GLN, GLRLM GLN-VN, GLSZM ZSN, GLSZM ZSV, Global Kurtosis, Prostate Volume
Sub-Acute	iCBCT SHARP VeryLow Llo 1	3	GLCM Contrast, GLCM Correlation, NGTDM Contrast
Sub-Acute	iCBCT SHARP VeryLow Uni 0	3	GLCM Correlation, GLRLM RLN, GLSZM ZSN
Sub-Acute	iCBCT STD Med Llo 1*	6	GLCM Contrast, GLCM Correlation, GLCM Dissimilarity, GLSZM LZLGE, NGTDM Coarseness, NGTDM Strength
Sub-Acute	iCBCT STD Med Uni 0	5	GLSZM GLN, GLSZM LZHGE, GLSZM LZLGE, GLSZM ZSV, NGTDM Coarseness
Sub-Acute	sCBCT SHARP Llo 1	4	GLCM SumAverage, GLRLM LRHGE, GLSZM GLN, GLSZM LZHGE
Sub-Acute	sCBCT SHARP Uni 0	5	GLCM Correlation, GLRLM GLV, GLSZM GLV, GLSZM ZSV, Global Skewness
Sub-Acute	sCBCT STD Llo 1	7	GLCM Variance, GLRLM RLN, GLSZM GLN, GLSZM LZLGE, Global Kurtosis, NGTDM Contrast, NGTDM Strength
Sub-Acute	sCBCT STD Uni 0*	4	GLRLM RLN, GLSZM ZSN, Global Skewness, NGTDM Coarseness-VN
∆IPSS	iCBCT SHARP VeryLow Llo 1	6	GLRLM GLN, GLRLM LRLGE, GLRLM RLN, GLSZM ZSN, IPSS Baseline, NGTDM Contrast
∆IPSS	iCBCT SHARP VeryLow Uni 0	4	GLRLM RLN, GLSZM SZE, IPSS Baseline, NGTDM Coarseness
∆IPSS	iCBCT STD Med Llo 1	4	GLRLM RLN, GLRLM RLV, GLRLM SRE, IPSS Baseline
∆IPSS	iCBCT STD Med Uni 0*	4	GLRLM RLN, GLSZM SZLGE, GLSZM ZP, IPSS Baseline
∆IPSS	sCBCT SHARP Llo 1*	4	GLRLM GLN, GLRLM RLN, Global Variance, IPSS Baseline
∆IPSS	sCBCT SHARP Uni 0	3	GLRLM RLN, IPSS Baseline, NGTDM Contrast
∆IPSS	sCBCT STD Llo 1	6	GLCM Variance, GLRLM GLN, GLRLM RLN, GLSZM LZE, Global Variance, IPSS Baseline
∆IPSS	sCBCT STD Uni 0	6	GLCM Correlation, GLRLM RLN, GLSZM GLN-VN, IPSS Baseline, NGTDM Coarseness, NGTDM Contrast

### Acute GU toxicity

The comparison of Acute GU toxicity delta-radiomics model performance for different reconstruction and preprocessing methods is summarized in Fig. 5. The range of median AUC values for the iCBCT-based models of Acute GU toxicity was between 0.64 and 0.71 in the box and whisker plot shown in Fig. 5. The range of median AUC values for the sCBCT-based models of acute GU toxicity was between 0.69 and 0.74. While there was more variation for iCBCT-based models than the sCBCT-based models for the different reconstruction and preprocessing methods considered, a similar noticeable drop in AUC can be seen for both iCBCT and sCBCT when comparing no Collewet normalization (0) to with Collewet normalization (1). Figure 5, for example, shows that the median AUC is equal to 0.71 for iCBCT-Std-Medium without Collewet normalization and equal to 0.64 for iCBCT-Std-Medium with Collewet normalization. The median AUC drops 0.07 for iCBCT-Std-Medium with the application of Collewet normalization. For sCBCT, the median AUC drops by a smaller magnitude. The reconstruction and preprocessing methods of iCBCT-Std-Med-Uni-0 and sCBCT-Sharp-Uni-0 showed the smallest spread and highest median AUC in their respective categories over all the BED bins and bootstrapping iterations.

The performance of the delta-radiomics models for the final selected Acute GU toxicity as function of BED is shown in Fig. 6. The sCBCT-based model for acute GU toxicity consistently has a median AUC greater than 0.7, while the iCBCT-based model falls below AUC of 0.7 between 40 and 80 Gy accumulated BED and otherwise is above 0.7 AUC. The AUC of the sCBCT-based models also drop slightly during the middle range of accumulated BED values. The AUC of the Acute GU toxicity models is highest after 100 Gy accumulated BED bins, though the Acute GU toxicity dataset starts to approach outcomes more in common with the Sub-Acute GU toxicity outcomes.

### Sub-acute GU toxicity

The comparison of Sub-Acute GU toxicity delta-radiomics model performance for different reconstruction and preprocessing methods is summarized in Fig. 7. The reconstruction and pre-processing methods with the largest median AUC were iCBCT-Std-Med-Llo-0 and sCBCT-Std-Uni-0 with median AUC equal to 0.78 and 0.83, respectively. The median AUC of the other iCBCT reconstruction and preprocessing methods were between 0.62 and 0.70. Consequently, the iCBCT-Std-Med-Llo-0 was a clear choice. For sCBCT, the choice for selected reconstruction and pre-processing method was remarkably close between sCBCT-Std-Llo-1 and sCBCT-Std-Uni-0. However, sCBCT-Std-Uni-0 was chosen due to its larger median and smaller spread in values over all BED bins and bootstrap iterations.

The performance of the selected sub-acute GU toxicity delta-radiomics models versus accumulated BED is shown in Fig. 8. A notable difference between the iCBCT-based model and sCBCT-based model is that the iCBCT-based models often have a larger spread values which can be seen through the spread in the interquartile range, the box. The spread of the minimum and maximum values, the whiskers, are also larger for iCBCT-based models. For both the iCBCT and sCBCT based models, the sub-acute GU toxicity there appear to be two local maxima in the AUC. For iCBCT models, the maximum AUC BED points are 40 and 80 Gy, while for the sCBCT they are 40 and 100 Gy. The highest AUC corresponds to sCBCT models that achieve AUC greater than 0.88 for the 40 and 100 Gy BED bins. The sCBCT models more often produced AUC greater than 0.8 than the iCBCT models for sub-acute GU toxicity.

### ∆IPSS

The comparison of ∆IPSS delta-radiomics model performance for different reconstruction and preprocessing methods is summarized in in Fig. 9. In comparison to the GU toxicity models in the previous sections, the ∆IPSS models show less variation between the iCBCT and sCBCT-based models. The selected reconstruction and pre-processing methods for ∆IPSS were iCBCT-Std-Med-Uni-0 (0.73 median AUC) and sCBCT-Sharp-Llo-1 (0.77 median AUC) for iCBCT and sCBCT, respectively.

The performance of the selected ∆IPSS delta-radiomics models versus accumulated BED is shown in Fig. 10. Both the iCBCT and sCBCT delta-radiomics models were greater than 0.7 AUC. With an AUC of 0.83, the sCBCT showed strong performance after 20 Gy accumulated BED but decreases thereafter. For iCBCT, the ∆IPSS model AUC oscillates between 0.76 and 0.71 for the various BED bins throughout the course of treatment.

## Discussion

The aim of this pilot study was to test the feasibility of creating radiomic models of sufficient quality to predict acute toxicity, sub-acute GU toxicity, and ∆IPSS from quantitative radiomic features extracted from daily CBCT images acquired during definitive RT of PCa. This study found strong evidence supporting the predictive potential of CBCT-based delta-radiomics. In comparison to other imaging modalities like diagnostic MRI and planning CT, CBCT images suffer from poorer image quality. While several CBCT-based delta-radiomics studies have been published in the past, this approach represents a novel strategy for PCa by using CBCT images^29,44,55–59^. Moreover, previous studies in other cancer sites have shown that CBCT-based delta-radiomics is viable for building predictive models^29,44,55–59^. In this work, we have demonstrated that moderately performing models (AUC > 0.7) were possible for acute and sub-acute GU Toxicity as early as the initial 20 Gy of accumulated BED, which translates to the first week of definitive RT. The ∆IPSS model had greater than 0.85 median AUC based on our internally validated test using the leave one out analysis with 1000 iterations bootstrapping.

This study sought to keep image parameters as consistent as possible while also achieving sufficient patient sample size to develop models with meaningful statistical power. A unique feature of this study is the collection of raw projection data from daily CBCT scans, which allowed for retrospective image reconstructions with various parameters. Parallel data sets were generated from the raw projection CBCT data using both standard back-projection reconstruction and iterative-based reconstruction. It was hypothesized that the iterative-based techniques would improve image quality and thus produce better-performing models.

sCBCT-based models often outperformed iCBCT-based models for the three clinical endpoints. This can be seen in Figs. 6, 8, and 10. In the box and whisker plots, sCBCT-based models often had a higher median AUC and lower spread than the iCBCT-based models. At first glance this appears counter-intuitive since iterative-based algorithms should have lower noise and higher image quality. The iterative reconstruction algorithm could be filtering out fine textural details important for quality model building. While iteratively reconstructed CBCT images are more appealing to the eye and improve contrast, in the process of smoothing the image the algorithm may remove clinically relevant information. Previous studies have shown that an iterative reconstruction algorithm with low noise suppression increases radiomic feature repeatability^31^. Analysis of the modulation transfer function of CT images has shown that images reconstructed with an iterative algorithm had more attenuation in the high spatial frequency portion of the spectrum than standard back-projection reconstruction algorithms^60,61^. In effect, the iterative reconstruction algorithm acts like a low pass filter in spatial frequency. It follows that less high spatial frequency information transits the iterative reconstruction, desirable for noise minimization. However, other high spatial frequency information and finer image details may be unintentionally lost as a result. In our study, the three clinical models all related in some fashion to GU toxicities. Early onset inflammation of prostate and urethral tissue due to RT may manifest in subtle textural changes that the iterative reconstruction algorithm effaces, leading to less effective prediction of subsequent acute and sub-acute adverse events.

The feature preprocessing method applied to CBCT images and cumulative BED impacted model performance. The effect of the preprocessing method was variable depending on the clinical endpoint: For the sub-acute models, as shown in Fig. 7, applying Llo-1 pre-processing sometimes increased AUC in comparison to Uni-0, while at other times it decreased AUC or had no effect. The role of feature pre-processing in AUC is confounded by the selection of different radiomic features for different reconstruction algorithms, as can be seen in Table 2. For the ∆IPSS models, other than iCBCT-Std, all reconstruction algorithms have smaller AUC for Uni-0 than Llo-1, as seen in Fig. 9. However, differences due to preprocessing are very small for the ∆IPSS models. In contrast, for all the Acute GU toxicity models shown in Fig. 5, Llo-1 had a lower median AUC than Uni-0. The decreased performance seen in the Llo-1 models of Acute GU toxicity could be due to the application of the Collewet normalization, indicated by the "1". Collewet normalization is a method used to remove outliers from the ROI and is commonly used in MRI-based radiomics studies^33^. It could be that the application of Collewet normalization leads to a loss in the intensity bias of the image which may contain vital information for model building.

Model performance varied throughout the course of treatment, as noted in Figs. 6, 8, and 10. For the acute and sub-acute GU toxicity models there is a dip in model performance midway through treatment, more apparent in acute than in the sub-acute models. Often model performance then improved in either of the final two accumulated BED bins (100 and 120 Gy). Within the scope of this pilot study and this sample size, we can only hypothesize the reason for the improvement in model performance towards the end of treatment. There could be a temporal pattern to toxicity. Patients who experience significant inflammation during the first week are likely to develop subsequent acute and subacute symptoms, and patients who develop inflammation toward the end of treatment due to the cumulative effect of radiation are also likely to develop subsequent GU symptoms. In between these 2 temporal peaks, the impact of prostate inflammation on eventual toxicity is harder to interpret. The best ∆IPSS model (sCBCT-Std-Llo-1) starts out better performing, decreases in performance in the middle, and stabilizes towards the end of treatment, as shown in Fig. 10. One notable difference in the ∆IPSS model is the use of the baseline IPSS as a covariate in the model. The baseline IPSS has been shown to be a strong predictor of post-treatment IPSS^62,63^. As time progresses and more BED is accumulated to the patient, the difference in time increases from when the baseline IPSS was acquired to when the CBCT images were acquired. Consequently, it could be that the baseline IPSS becomes more and more irrelevant to the model and leading to a decrease in model predictive performance as a result.

Limitations of this study included the presence of image artifacts in daily CBCT, image quality limitations, lack of independent validation, use of different dose fractionations, and patient sample size limitations. Most prostate patients in this cohort had gold fiducial markers implanted in the prostate prior to RT, resulting in streak artifact within the ROI on CBCT images. A gold fiducial artifacts algorithm developed at our institution was used to remove streak artifact from the prostate ROI, allowing for 3D radiomics. Other metal artifact strategies could be explored, like an iterative algorithm that removes artifacts directly in the raw projections. However, that technology is not yet available at our institution and is beyond what can be achieved in the scope of this study.

The limitations of small patient sample size can be overcome over time. However, a challenge is the time spent adjusting the contours on the daily CBCT images. A larger patient cohort will make it difficult to replicate the results of this study due to time costs. Auto segmentation of the prostate contours through the use of artificial intelligence, deep learning, or deformable propagation could greatly reduce the time it takes run radiomics studies such as this^42^. Future work will investigate performance of automated contours in comparison to manually segmented contours for radiomic analysis. One interesting finding of this study was that sCBCT-based delta-radiomics performed well. Most patients were scanned daily with CBCT using standard reconstruction. Consequently, it may be possible to greatly increase patient sample size for model building in future studies by using patients in our database before iCBCT was implemented in the clinic.

Another limitation of this study is the use of different dose fractionation schemes. To approach an adequate patient sample size this study binned the DRF based on the accumulated BED, which ultimately is a biologically relevant quantity. The use of different dose fractionation may introduce variables in the patient data set. In an internal analysis we found that the models produced using just one dose fractionation were not significantly changed with the addition of the patients who had 26 fractions. Most patients in this study were treated with very similar dose fractionations. 13 patients were treated on 26 fraction schedules and the remaining 36 patients were treated with greater than 37 fraction, as shown in more detail in Supplementary Table S1. A future study with a larger cohort could further explore the use of different dose fractionations on DRF.

This study focused on using the CBCT raw projection files to allow for retrospective assessment of different reconstruction algorithms and only 50 patients could be included for analysis. In addition, we opted not to include patients subjected to different imaging parameters and CBCT imaging modalities over the years, further limiting the sample size available for analysis. It remains to be seen if the analysis presented in this project will be generalizable to other CBCT imaging modalities and parameters.

Future studies will consider different patient outcomes, accumulate more patients, include an independent validation, overcome time inefficiency in the workflow, and provide a clear biologic rationale to explain the predictive power of radiomic features. PCa has a long natural history with treatment response and toxicity manifesting after many years. Many patients in this study are only 2 years post-RT, before clinical outcomes have matured. Other endpoints worthy of consideration for future analysis include intraprostatic persistence and recurrence, late toxicity, biochemical control, distant metastasis, and overall survival. This work focused on acute and subacute GU toxicities and IPSS since they are documented relatively early in the arc of treatment and follow up. As this field further develops, we hope to gain a better understanding of the biological changes underpinning these predictive delta-radiomics features.

## Conclusion

This pilot study analyzed the performance of CBCT-based delta-radiomics of prostate cancer to predict acute and subacute GU toxicity and ∆IPSS. As early as the first 20 Gy BED, corresponding to the first week of RT, CBCT-based delta-radiomics features predicted acute and subacute GU toxicity and ∆IPSS with moderate performance (AUC > 0.7). Two acute GU toxicity models had AUC > 0.8. Three sub-acute GU toxicity models had AUC > 0.88. The ∆IPSS model had the strongest performance for the 20 Gy BED bin with a median AUC equal to 0.83. Early warning of potential RT toxicities can prompt interventions that may prevent or mitigate future adverse events^18,19^.

## Supplementary Information

Supplementary Information.

## Data Availability

The datasets generated and/or analyzed during the current study are not publicly available due to being collected as part of an ongoing clinical trial. Clinical trial relevant data will be made public upon completion of accrual and publication of the primary endpoint. Contact the corresponding author, N.D, to request data from this study.
