# Supplementary material for: Cone-beam CT delta-radiomics to predict genitourinary toxicities and international prostate symptom of prostate cancer patients: a pilot study
Source: Sci Rep. 2022 Nov 22;12:20136. doi: 10.1038/s41598-022-24435-8 (PMC9684516; doi:10.1038/s41598-022-24435-8)
Supplement: Supplementary file 1 — Supplementary Information. [file 41598_2022_24435_MOESM1_ESM.pdf]

Supplementary Information for "Cone-beam CT Delta-radiomics to Predict Genitourinary Toxicities and International Prostate Symptom of Prostate Cancer Patients: A Pilot Study"

1. Supplementary Tables

Supplementary Table S1. Patient characteristics.

Characteristics	Median	Range
Age (yr)	50	52-87
Gross Tumor Volume (cm^3)	47.2	16-111
	Number of Patients	Percent of Patients (%)
Gleason Score		
3+3	8	16.0
3+4	14	28.0
4+3	7	14.0
4+4	4	8.0
4+5	14	28.0
5+4	1	2.0
5+5	1	2.0
Missing	1	2.0
Tumor Stage		
T1c	18	36.0
T2a	3	6.0
T2b	8	16.0
T2c	4	8.0
T3a	9	18.0
T3b	2	4.0
Missing	6	12.0
Hormonal Use		
Used	37	74.0
None	13	26.0
Total dose / Total fractions		
70.2 Gy / 26 fx	13	26.0
74 Gy / 37 fx	1	2.0
76 Gy / 38 fx	5	10.0
80 Gy / 40 fx	26	52.0
81.4 Gy / 37 fx	1	2.0
86 Gy / 40 fx	1	2.0
91.2 Gy / 38 fx	3	6.0

Supplementary Table S2. Summary of percentage of events by outcome and BED level.

Outcome	BED [Gy]	Category 0	Category 1
Acute GU	20	28%	72%
Acute GU	40	29%	71%
Acute GU	60	33%	67%
Acute GU	80	31%	69%
Acute GU	100	32%	68%
Acute GU	120	37%	63%
Sub-acute GU	20	44%	56%
Sub-acute GU	40	44%	56%
Sub-acute GU	60	44%	56%
Sub-acute GU	80	44%	56%
Sub-acute GU	100	44%	56%
Sub-acute GU	120	44%	56%
ΔIPSS	20	60%	40%
ΔIPSS	40	60%	40%
ΔIPSS	60	60%	40%
ΔIPSS	80	60%	40%
ΔIPSS	100	60%	40%
ΔIPSS	120	60%	40%

Supplementary Table S3. Forty-two radiomic features were considered in this study. IBSI codes listed in brackets.

Feature Class	Description	Features
Gray-level Co-occurrence Matrices (GLCM) [LFYI]	GLCM feature used voxel displacements of 1 to quantify the frequency of a pattern of two intensities occurring together throughout an image.	Contrast [ACUI]<br>Correlation [NI2N]<br>Dissimilarity [8S9J]<br>Energy [8ZQL]<br>Entropy [TU9B]<br>Homogeneity [IB1Z]<br>Sum Average [ZGXS]<br>Variance [UR99]
Gray-level Run Length Matrices (GLRLM) [TP0I]	GLRLM features encode for different run lengths of connected isotone voxels. Runs are computed in 13 directions of three-dimensional space.	Gray-level Non-Uniformity (GLN) [R5YN]<br>Gray-level Variance (GLV) [8CE5]<br>High Gray-level Run Emphasis (HGRE) [G3QZ]<br>Long Run Emphasis (LRE) [W4KF]<br>Long Run High Gray-level Emphasis (LRHGE) [3KUM]<br>Long Run Low Gray-level Emphasis (LRLGE) [IVPO]<br>Low Gray-level Run Emphasis (LGRE) [V3SW]<br>Run Length Variance (RLV) [SXLW]<br>Run Percentage (RP) [9ZK5]<br>Run-Length Non-Uniformity (RLN) [W92Y]<br>Short Run Emphasis (SRE) [220V]<br>Short Run High Gray-level Emphasis (SRHGE) [GD3A]<br>Short Run Low Gray-level Emphasis (SRLGE) [HTZT]
Gray-level Zone Size Matrices (GLZSM) [9SAK]:	GLSZM features quantify image textures by considering the frequency of occurrence of all isotone gray-level regions	Gray-level Non-Uniformity (GLN) [JNSA]<br>Gray-level Variance (GLV) [BYLV]<br>High Gray-level Zone Emphasis (HGZE) [5GN9]<br>Large Zone Emphasis (LZE) [48P8]<br>Large Zones High Gray-level Emphasis (LZHGE) [J17V]<br>Large Zones Low Gray-level Emphasis (LZLGE) [YH51]<br>Low Gray-level Zone Emphasis (LGZE) [XMSY]<br>Short Zone Emphasis (SZE) [5QRC]<br>Short Zones High Gray-level Emphasis (SZHGE) [HW1V]<br>Short Zones Low Gray-level Emphasis (SZLGE) [5RAI]<br>Zone Percentage (ZP) [P30P]<br>Zone Size Non-Uniformity (ZSN) [4JP3]<br>Zone Size Variance (ZSV) [3NSA]
Neighborhood Gray-Tone Difference Matrix (NGTDM) [IPET]	NGTDM features quantify the difference between a voxel of interest and average of the surrounding 3 × 3 × 3 voxel neighborhood	Busyness (BUSY) [NQ30]<br>Coarseness (COAR)*<br>Complexity (CPLX) [HDEZ]<br>Contrast (CONT) [65HE]<br>Strength (STRG)*
Intensity-based Statistics (IS) [UHIW]	Statistics of region of interest.	Kurtosis [IPH6]<br>Skewness [KE2A]<br>Variance [ECT3]

*NGTDM-based coarseness and strength are calculated as defined by Amadasun and King[43].

2. Supplementary Figures

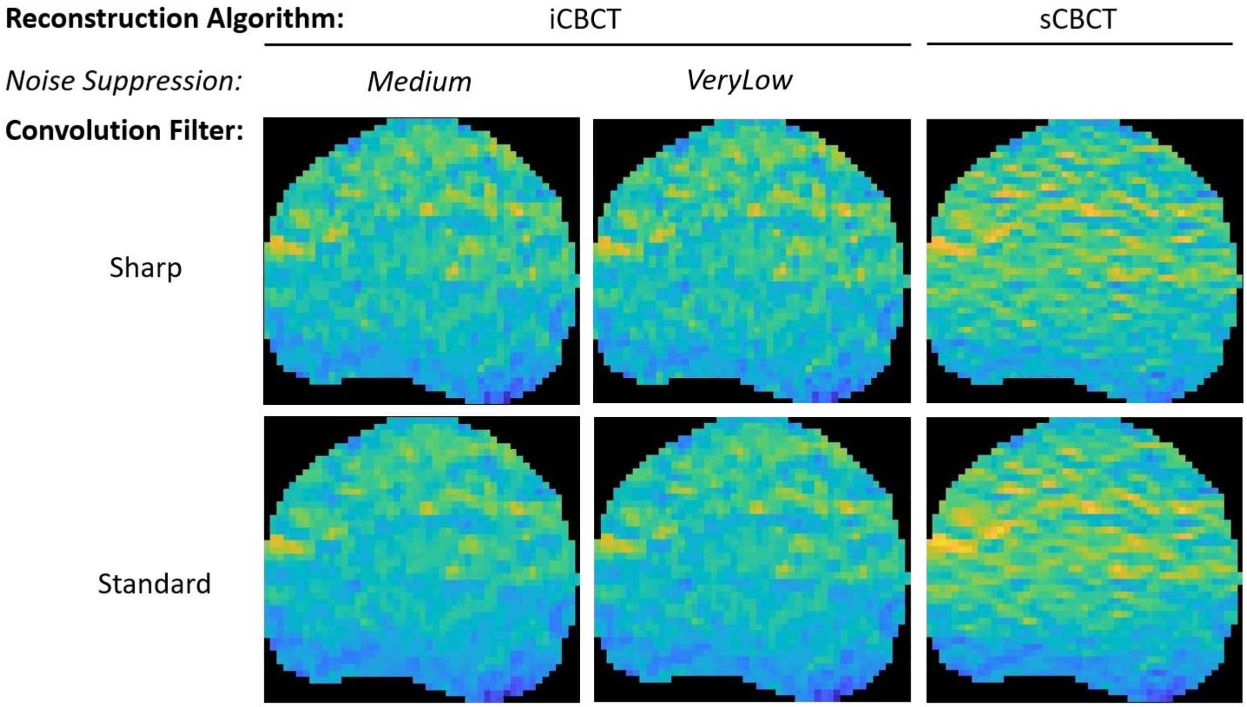

Supplementary Figure 1: Reconstructed CBCT images of a prostate cancer patient using various reconstruction methods considered in this work. This example shows the same image slice of the prostate for one patient that has been reconstructed with different reconstruction methods.

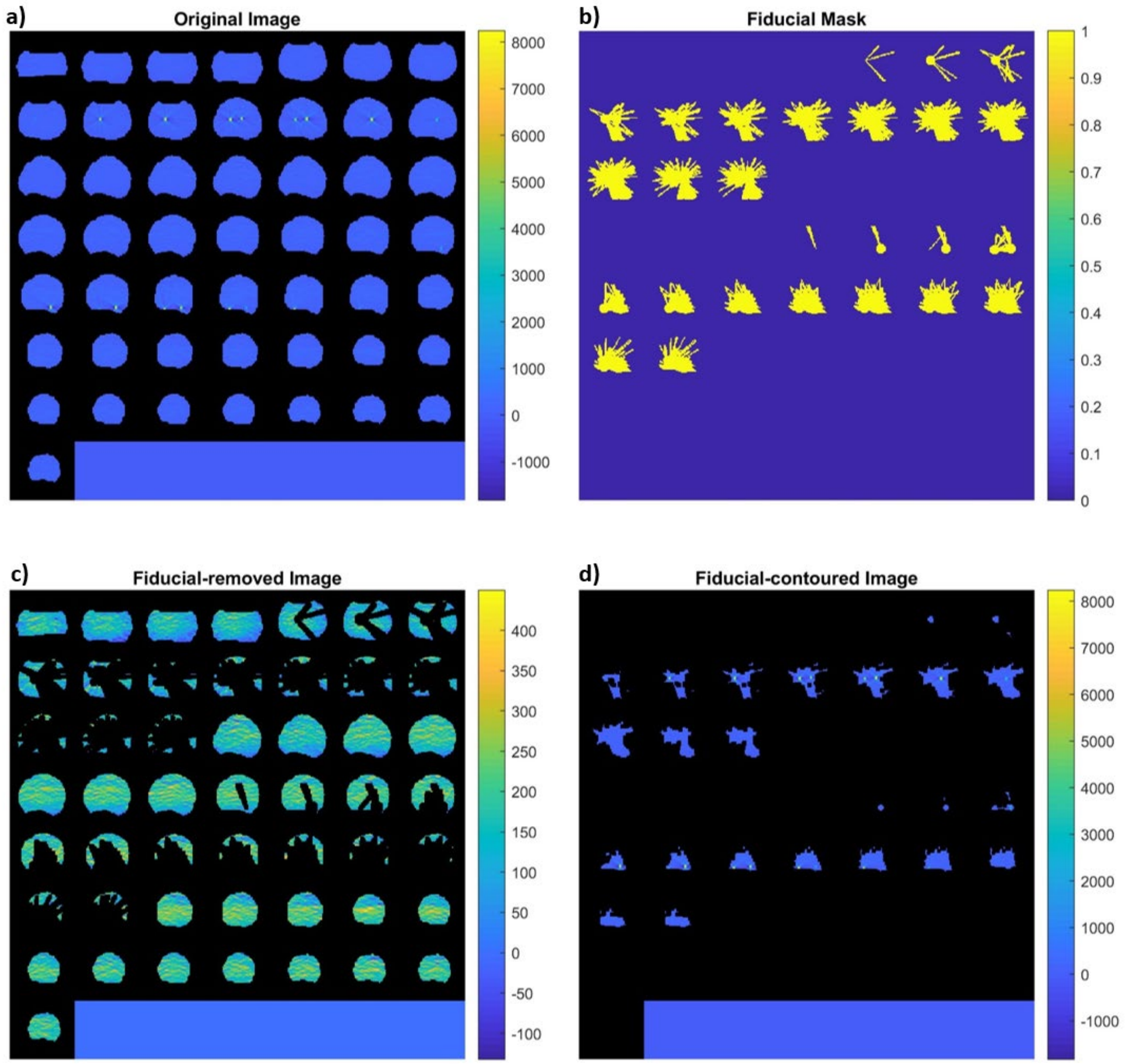

Supplemental Figure 2: Example of gold artifact removal algorithm of a prostate CBCT image showing the different stages of the process that include a) the original image, b) the fiducial mask, c) fiducial-removed image, and d) the fiducial portion of the image that was removed.
